# Laparoscopic staging in gastric cancer: An essential step in its management

**DOI:** 10.4103/0972-9941.72597

**Published:** 2010

**Authors:** D Mahadevan, A Sudirman, P Kandasami, G Ramesh

**Affiliations:** Department of Surgery, Division of Upper GI Surgery, Tuanku Jaafar Hospital, Seremban, Malaysia

**Keywords:** Gastric cancer, laparoscopic staging

## Abstract

**AIM:**

The role of laparoscopy in staging of gastric cancer is widely accepted; however, in Malaysia its usage has been limited. Patients can be classified as resectable or unresectable, which helps in avoiding an unwanted laparotomy and the morbidities associated with it. The aim of this study was to assess the value of laparoscopy in staging of gastric cancer in comparison with CT scan.

**MATERIALS AND METHODS:**

Patients with carcinoma of the stomach after a complete preoperative work-up underwent laparoscopy prior to surgical exploration. TNM staging was used to compare laparoscopy with CT, with the histopathological report used as the gold standard.

**RESULTS:**

Forty cases were included in this study. The sensitivity of laparoscopy for T3 tumours appears to be significant when compared to that of CT. Laparoscopy detected 90.3% of the cases as against the 58% detected with CT. There was not much difference in the N factor. With regard to M factor, the sensitivity was 100% for laparoscopy in comparison with CT.

**CONCLUSIONS:**

Laparoscopy has been shown to be sensitive in detecting metastasis in gastric cancer in comparison to CT, thus helping in avoiding unwanted laparotomy and thus providing a more systemic approach in managing gastric cancers.

## INTRODUCTION

Surgical resection continues to remain the only potentially curative treatment for patients with gastric cancer. However, surgical resection is dependent on the staging of the disease at laparotomy. It was reported by Kandasami *et al*., in 2003, that 82% of patients with gastric cancer presented with stage IV disease, and curative surgery was offered only to 16% of them.[1] In a substantial number of patients, not even a palliative procedure was offered. Vistre *et al*., in 1988, reported that 25% of patients with gastric cancer underwent unnecessary laparotomy, and 13% to 23% developed complications due to the laparotomy.[2] Accurate preoperative staging can help reduce the number of unnecessary surgeries and decide on other options of treatment. Computed tomography is a routine preoperative investigative work-up and most often under-stages the disease.[3] The high false-negative rate for CT scans has been shown by prospective studies,[4] as CT scan does not totally exclude liver and peritoneal metastasis.

Endoscopic ultrasound is not routinely used for staging gastric cancer in Malaysia due to its limited availability. Laparoscopic staging has the ability to assess peritoneal and liver metastasis, thus helping in avoiding unnecessary laparotomy for advanced cases. Several recent studies have demonstrated that laparoscopic staging may substantially reduce the need for exploratory laparotomy.[5] Peritoneal metastasis compromises gastric resection and is associated with poor survival.[6] In this study, we evaluate the usefulness of routine preoperative laparoscopic staging to decide on the resectability of gastric cancers.

## MATERIALS AND METHODS

A prospective study was carried out from 2006 to 2008 to assess the value of preoperative laparoscopic staging for gastric cancer. Patients with proven adenocarcinoma of the stomach after diagnostic work-up were included. Patients with obvious unresectable disease, e.g., liver metastasis, ascites, on CT scan were excluded. A total of 40 patients were included in this study. Patients underwent laparoscopic staging under general anaesthesia. After the staging, if deemed resectable, we proceeded with laparotomy. The patients are placed in supine position, and a 12-mm trocar is inserted into the subumbilical region. A telescope angled at 30° is used to inspect the peritoneal cavity. Another trocar, 5 mm, is inserted into the left subcostal region to assist in lifting the stomach to assess mobility and to inspect more thoroughly under the liver surface. First the site of the tumour is assessed, and its T stage is assessed. Then we proceed to inspect the liver, which includes its outer surface and below, to look for secondaries. Then we proceed to inspect the peritoneum for metastasis; and finally, the pelvis. We routinely do not enter the lesser sac for inspection as locally advanced tumours adherent to pancreas make mobilization more difficult in this region. We also do not have facilities for laparoscopic ultrasound in most hospitals in Malaysia. If suspicious lesions are found in the peritoneum or if ascitic fluid is detected, specimens are taken for histology and cytology to exclude metastasis. If there are suspicious lesions, we do not proceed for resection till confirmatory results are available. If lesions are proven not to be metastatic, then surgery is performed when the next operation is scheduled.

Laparoscopic staging is performed by assessing for evidence of peritoneal, liver metastasis (M+) or ascites. The T factor is assessed by looking for serosal involvement or involvement of adjacent organs; and the N factor, by involvement of adjacent lymph nodes.

Pathological examination was considered as the gold standard for the staging. This was compared with the CT and laparoscopy with regard to efficacy of staging. The CT scan used is a 16-slice scanner, and patients are given oral and intravenous contrast prior to examination.

## RESULTS

A total of 40 patients underwent laparoscopic examination during the period of this study. Mean age of the patients was 60 years. There were 28 males and 12 females. Forty cases were included in this study; and the T factor, M factor and N factor were analyzed — histopathological report being the gold standard. The results with regard to T factor are shown in Table 1. Although statistically not significant with regard to the T factor, the sensitivity of laparoscopy for T3 tumours appears to be significant when compared to that of CT. Laparoscopy detected 90.3% of the cases as against the 58% detected with CT.

**Table 1:** T staging

	hpe	CT	Lap.	P
T2	4	3 (75)	2 (50)	0.6370
T3	31	18 (58.1)	28 (90.3)	Not
T4	5	2 (40)	4 (80)	significant
Overall	40	23 (57.5)	34 (85)	
Total	40	23	34	

Sensitivity CT: Overall: 57.5%; T2: 75%; T3: 58.1%; T4: 40%; Sensitivity Lap.: Overall: 85%; T2: 50%; T3: 90.3%; T4: 80%

With regard to N factor [Table 2], there was not much difference between laparoscopy and CT. Laparoscopy had a sensitivity of 70%; and CT, 62.5%.

**Table 2:** N staging

	Positive finding	Negative finding	
CT	25	15	40
Lap.	28	12	40

n=40; Sensitivity CT: 62.5%. Sensitivity Lap.: 70%.

The most significant finding in this study was with regard to the M factor. All patients were staged at M0 before laparoscopy. Laparoscopy detected 7 cases of peritoneal metastasis, which was not seen on CT. Among these 7 patients, 3 had suspicious peritoneal lesions, which were then confirmed on histology. In all these patients, we avoided an unnecessary laparotomy. The rate of unnecessary laparotomies would have been 17% among these 40 cases if a laparoscopic staging was not done. The sensitivity was 100% for laparoscopy, which indicates laparoscopy is the diagnostic choice for detecting peritoneal metastasis. CT scan did not show any distant metastasis in any of the 40 cases; but laparoscopy picked up 7 (M+) cases, which were missed on CT [Table 3]. Patients with no metastasis on laparoscopy had potentially curative surgery done for them. All of these 7 patients benefited from not having to undergo a laparotomy and only underwent laparoscopy as intervention as they had no obstruction warranting a palliative bypass surgery and had adjuvant therapy.

**Table 3:** M staging

	M0	M+	Sensitivity (%)	Total	P
CT	33/40	-	82.5	40	0.0117
Lap.	33/33	7/7	100	40	

## DISCUSSION

Staging for gastric cancer prior to surgery is very important in assessing outcomes. Despite the introduction of CT scanning and magnetic resonance imaging, imaging methods lack sufficient accuracy when dealing with gastric cancer. Avoiding any unnecessary laparotomy should be of importance. Staging of this disease enables us to categorize patients into a curative or palliative group. It is well known that exploratory laparotomy for confirmation of unresecta bility has its disadvantages. It has been reported that complications occur in 12% to 23% of unresectable patients, with mortality ranging from 10% to 21.1%.[2]

In our study, we showed that for M disease, laparoscopy was very sensitive in detecting metastasis. Possik *et al*. reported sensitivity of 83% in the detection of peritoneal metastasis and 87% in the detection of liver metastasis in a series of 360 cases.[7] Gretschel *et al*. showed the sensitivity for detecting peritoneal metastasis with laparoscopy was 85% as compared to 28% with CT.[8] Gross *et al*. evaluated 46 consecutive patients with adenocarcinoma of the stomach; and among them, laparoscopy identified 27 cases of metastatic disease.[9] They concluded laparoscopy can avoid unnecessary exploration and is effective in directing therapy. Lehnert *et al*. obtained encouraging results with laparoscopy and found the procedure to be well tolerated.[10] We too in our study showed that we can avoid unnecessary laparotomy, the rate of which would have been about 17% in our series of 40 patients. On the basis of laparoscopy, all these patients were spared surgery, and the morbidities associated with surgery were avoided. Historically, prior to the introduction of laparoscopic staging, the rate of unnecessary laparotomy in our institution was high due to improper staging.

The ability to assess the T factor is quite variable. T3 tumours are easily detected laparoscopically, probably due to the serosal involvement. This was shown in our study. Lesions < T3 are more difficult to assess laparoscopically. The ability to assess the N factor too did not show any significant difference when laparoscopy was compared to CT. With the introduction of laparoscopy in staging of gastric cancer, an algorithm of management of gastric cancer can be applied. Patients detected with T3N1M0 tumours can be considered suitable for neoadjuvant chemotherapy, and those detected to have metastasis can be considered suitable for palliative care. McCulloh *et al*.[11] showed in their study that the impact of laparoscopy on clinical decision making was substantial, altering previous management plan in 34% of the cases. Laparoscopy is also shown to be beneficial in obtaining peritoneal fluid for cytology to detect metastasis.

At our centre, we also find the usefulness of laparoscopy under general anaesthesia as a stress test for cardiorespiratory assessment prior to major exploration. We feel, in view of its major role in the assessment and planning of management of gastric cancer, laparoscopy should be an integral part of preoperative staging of gastric cancer. Laparoscopic staging for gastric cancers also plays an integral role in avoiding unnecessary laparotomies in patients with advanced disease.

## CONCLUSION

Laparoscopic staging at our centre has been shown to improve the management and staging of gastric cancers, thus providing the best treatment possible taking into consideration the stage of the disease; it has also been shown to reduce unwanted laparotomies.
